# Improved tractography using asymmetric fibre orientation distributions

**DOI:** 10.1016/j.neuroimage.2017.06.050

**Published:** 2017-09

**Authors:** Matteo Bastiani, Michiel Cottaar, Krikor Dikranian, Aurobrata Ghosh, Hui Zhang, Daniel C. Alexander, Timothy E. Behrens, Saad Jbabdi, Stamatios N. Sotiropoulos

**Affiliations:** a Wellcome Centre for Integrative Neuroscience (WIN) - Oxford Centre for Functional Magnetic Resonance Imaging of the Brain (FMRIB), University of Oxford, UK; b Department of Neuroscience, Washington University, St. Louis, MO, USA; c Department of Computer Science & Centre for Medical Image Computing, University College London, UK; d Sir Peter Mansfield Imaging Centre, School of Medicine, University of Nottingham, UK

**Keywords:** Diffusion MRI, Tractography, Structural connectivity, Asymmetry, Connectome

## Abstract

Diffusion MRI allows us to make inferences on the structural organisation of the brain by mapping water diffusion to white matter microstructure. However, such a mapping is generally ill-defined; for instance, diffusion measurements are antipodally symmetric (diffusion along **x** and –**x** are equal), whereas the distribution of fibre orientations within a voxel is generally not symmetric. Therefore, different sub-voxel patterns such as crossing, fanning, or sharp bending, cannot be distinguished by fitting a voxel-wise model to the signal. However, asymmetric fibre patterns can potentially be distinguished once spatial information from neighbouring voxels is taken into account. We propose a neighbourhood-constrained spherical deconvolution approach that is capable of inferring *asymmetric* fibre orientation distributions (A-fods). Importantly, we further design and implement a tractography algorithm that utilises the estimated A-fods, since the commonly used streamline tractography paradigm cannot directly take advantage of the new information. We assess performance using ultra-high resolution histology data where we can compare true orientation distributions against sub-voxel fibre patterns estimated from down-sampled data. Finally, we explore the benefits of A-fods-based tractography using in vivo data by evaluating agreement of tractography predictions with connectivity estimates made using different in-vivo modalities. The proposed approach can reliably estimate complex fibre patterns such as sharp bending and fanning, which voxel-wise approaches cannot estimate. Moreover, histology-based and in-vivo results show that the new framework allows more accurate tractography and reconstruction of maps quantifying (symmetric and asymmetric) fibre complexity.

## Highlights

• A new comprehensive framework for both asymmetric fod estimation and tractography.
• Extension of classical CSD approaches applicable to single and multi-shell data.
• Validation using anatomically relevant fibre patterns derived from histology.
• Correct reconstruction of sub-voxel fanning polarities and sharp bends.

## 1 Introduction

Over the last decades, several developments in the field of magnetic resonance imaging (MRI) have allowed researchers to investigate the anatomical organisation of the brain in vivo and non-invasively at the macroscopic level. Amongst several MRI data acquisition techniques, diffusion MRI (dMRI) has shown great potential to probe the organisation of white matter and structural connection patterns of the brain at different scales (Bastiani and Roebroeck, 2015, Jbabdi et al., 2015, Mori and van Zijl, 2002, Tournier et al., 2011). The pattern of diffusion displacements of water within tissue can be used to probe the main axonal orientations, as water molecules tend to diffuse preferentially along axons. Measurements are, therefore, made sensitive to water diffusion along different orientations. One can then estimate, in each voxel, a fibre orientation distribution (fod) that encodes the fraction of fibre bundles oriented along different directions. Once all the voxel-wise fods have been successfully obtained, a tractography algorithm can be used to reconstruct structural connections between different (sub-)cortical areas.

Despite the evident success of this technology in localising major fibre bundles (e.g., Catani et al., 2012, Catani and Thiebaut de Schotten, 2008, Johansen-Berg and Behrens, 2006), limitations arise from the indirect mapping of diffusion measurements to fods. Tens of thousands of white matter axons may be contained within a single dMRI voxel, where fibres can bend very sharply (e.g., when entering the wall of a cortical gyrus), fan out (e.g., when entering the crown of a cortical gyrus) or converge (e.g. when projections from different cortical regions converge to the main body of the internal capsule). Given the limited resolution of typical dMRI acquisitions and the inherent antipodal symmetry of the sampled signal (measurements along directions **x** and –**x** are equal), all these different sub-voxel patterns cannot be distinguished when considering only voxel-wise measurements (Jbabdi and Johansen-Berg, 2011, Seunarine and Alexander, 2009, Tournier et al., 2011). For instance, fibres fanning out and fanning in will give rise to the same voxel-wise fod. However, spatial information can be helpful in these cases (Savadjiev et al., 2006). A sharp bend would comprise of a different spatial arrangement of fods than a fan or a crossing.

This idea of incorporating information from neighbouring voxels in the estimation of fibre patterns has been proposed before. The motivation comes from the principle of fibre continuity (Reisert et al., 2012, Reisert and Kiselev, 2011) or co-helicity (Campbell et al., 2014, Savadjiev et al., 2006, Savadjiev et al., 2008); fibres should be continuous in space, therefore whatever leaves a voxel should enter one of its neighbours. Thus, a set of fods can be estimated using neighbourhoods of voxels. Using this principle, asymmetric functions have been estimated before, by spatially post-processing and regularising symmetric fods (Barmpoutis et al., 2008, Cetin et al., 2015, Ehricke et al., 2011) or by using specific geometric priors (Reisert et al., 2012, Savadjiev et al., 2006). However, there have been very few attempts to take advantage of the extra information provided by asymmetric fods in a tractography algorithm. Expanding the currently available tracking methods is not trivial (Campbell et al., 2014, Rowe et al., 2013) and simply plugging asymmetric fods into a typical tractography paradigm will not work (Ehricke et al., 2011), as current tractography methods expect within-voxel symmetric orientation information.

In this work, we propose a direct estimation of asymmetric fods (A-fods) from dMRI data based on a spherical deconvolution approach that can infer sub-voxel patterns. Key to the estimation of A-fods is the addition of neighbourhood continuity components in the fitting. The model uses a set of symmetric and asymmetric basis functions to represent A-fods and naturally extends the non-parametric constrained spherical deconvolution framework for both single (Tournier et al., 2004, Tournier et al., 2007) and multi-shell data (Jeurissen et al., 2014). Spherical deconvolution has been very successful in estimating fibre crossings, but our proposed approach allows to further assess sharp bends and fibre dispersion, including fanning polarity. Importantly, we also propose a tractography algorithm that extends previous frameworks to make use of the estimated A-fods. We assess the increased accuracy obtained from the A-fod model and tractography algorithm using anatomically-realistic fibre patterns and tracking extracted from high resolution histology. Finally, we show examples using in vivo data, investigating the effect of fanning polarity on tractography and showing benefits in resolving connection patterns when asymmetry is considered. In the absence of ground truth in-vivo, we compare connectivity mapping estimates from our approach with measures of connectivity obtained using resting state functional MRI (rs-fMRI).

## 2 Theory

### 2.1 Asymmetric fibre orientation density functions

**Fig. 1:**
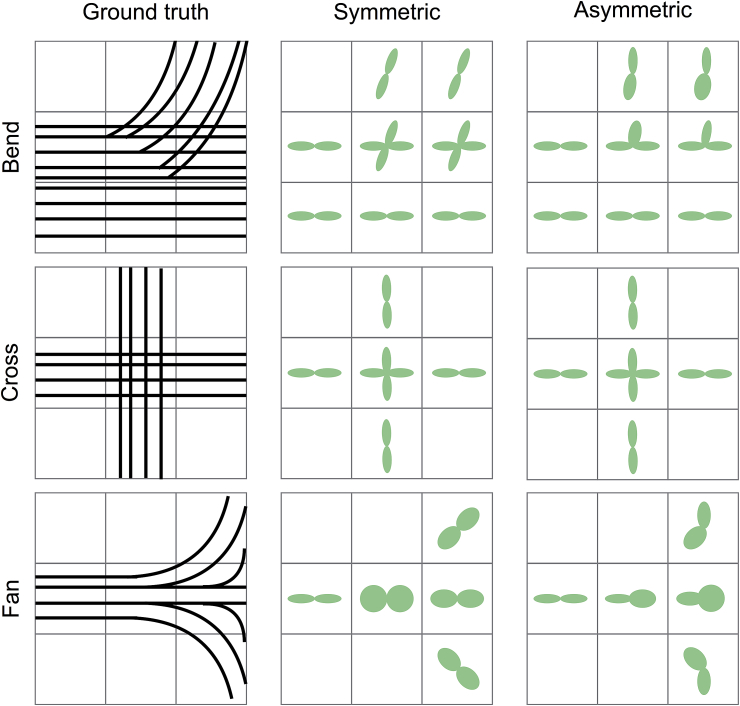
Toy examples of 3 different complex fibre patterns: sharp bend (top row), fibre crossing (middle row) and fibre fanning (bottom row). When using symmetric fods (middle column), sharp bending and fanning patterns cannot be accurately represented. When using asymmetric fods (rightmost column), only the meaningful fod peaks are selected both in the sharp bending and fibre crossing cases. Moreover, the right fanning polarity (i.e., increased dispersion along the left-right orientation) can be obtained.

The main motivation for our method is shown in the toy examples of Fig. 1. Even if the voxel-wise signal cannot always be uniquely predictive of the sub-voxel fibre orientations, the local spatial arrangement of orientations can be different depending on the nature of the voxel-wise pattern. Therefore, by considering information from the neighbourhood, we aim to resolve asymmetric fibre patterns.

Voxel-wise spherical deconvolution (SD) techniques for inferring fibre orientations assume that the dMRI signal *S* at every voxel is the convolution of a fibre orientation distribution *F* and a fibre response function *R*:

(1) S(θ,ϕ)=F(θ,ϕ)⊗R(θ),

where θ and ϕ are the elevation and azimuthal angles in spherical coordinates. The problem of determining *F* for a given response function *R* can be solved parametrically (Anderson, 2005, Behrens et al., 2007, Dell'acqua et al., 2010, Sotiropoulos et al., 2012) or non-parametrically (Descoteaux et al., 2007, Descoteaux et al., 2009, Jeurissen et al., 2014, Tournier et al., 2004, Tournier et al., 2007). An efficient and commonly-used non-parametric formalism for SD uses spherical harmonics (SH) (Descoteaux et al., 2007, Tournier et al., 2007), which form a linear orthonormal basis set over the unit sphere (see Supplementary material). Importantly, in the specific case of dMRI, the measurements are antipodally symmetric, and therefore only symmetric fods can be fitted to such measurements. This means that only even-order functions of the spherical harmonics basis can be considered. Asymmetric (i.e., odd-order) components can only capture noise and are therefore typically excluded from the estimation (Descoteaux et al., 2007, Tournier et al., 2007). This leads to the general formulation of SD as a constrained linear least squares problem:

(2) $$\hat{f}=arg\min_{f}{\left\|\mathrm{Cf}-Y\right\|}^{2},\;with\;\mathrm{Bf}\geq 0,$$

where ***f*** is a vector of unknown even-order coefficients, ***C*** is a matrix that encodes even-order basis functions convolved with the fibre response function, ***B*** is a matrix that maps the coefficients to the fod amplitudes on the sphere, and ***Y*** is the acquired dMRI signal in a voxel (see Supplementary material). The positivity constraint ensures that the fod amplitudes are always positive.

**Fig. 2:**
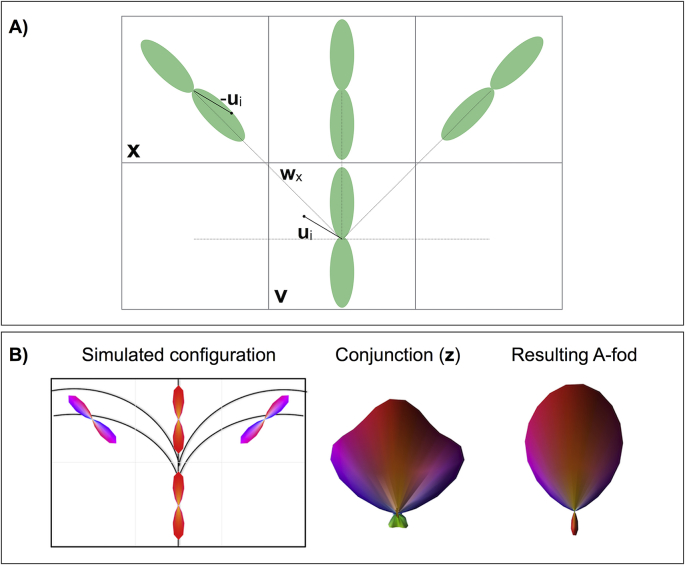
Asymmetric fod estimation procedure. A) General principle to obtain an A-fod in the current voxel *v* along orientation *u*_*i*_. When considering only one neighbouring voxel at a time, the algorithm tries to match the amplitude of f*od*_*v*_ along *u*_*i*_ with the one of *fod*_*x*_ along *–u*_*i*_. Voxel *x* and its *fod*_*x*_ are selected amongst the other neighbours of *v*, because the vector *w*_*x*_ (connecting the centre of voxel *v* to the one of voxel *x*, dashed line) is the closest to vector *u*_*i*_. B) Toy example of complex fibre pattern, i.e., fibre fanning. The first column shows the resulting voxel-wise S-fods overlaid on top of the simulated ground truth simulated patterns. The middle column shows the conjunction fod weighting the contribution of multiple neighbouring voxels (vector *z* in Eqs. (3), (4))) for the first iteration of the A-fod estimation algorithm (top right voxel in the bending case, leftmost voxel for the fibre fanning configuration). The last column shows that, after 3 iterations, the resulting A-fod captures the right fanning polarity (i.e., increased dispersion along the left-right orientation).

We extend the above framework by including the full spherical harmonics basis set in order to model asymmetric fods (A-fods). The conventional symmetric voxel-wise representation is augmented by incorporating information from neighbouring voxels. The proposed A-fod in a voxel is represented by both even and odd order SH functions. The even components are used to model the within-voxel signal, while the odd components allow for asymmetries informed by the spatial arrangement of the signal in neighbouring voxels. We devised the following cost function, minimised for the optimal set of SH coefficients to represent an A-fod:

(3) $$\hat{f_{\mathrm{all}}}=arg\underset{f_{\mathrm{all}}}{\min}\left\{{\left\|Cf_{\mathrm{even}}-Y_{v}\right\|}^{2}+\lambda^{2}{\left\|Bf_{\mathrm{all}}-z\right\|}^{2}\right\},\;with\;Bf_{\mathrm{all}}\geq 0$$

where ***f***_***all***_ contains both odd and even-order SH coefficients and ***f***_*even*_ contains only even order coefficients. As in Eq. (2), the first term minimizes the sum of squared residuals between the signal predicted by a voxel-wise symmetric fod and the acquired dMRI signal ***Y***_***v***_ for a given voxel ***v.*** The second term (weighted by the regularization parameter λ) minimizes the difference between the fod (***Bf***_*all*_) at that voxel and the conjunction of fods' amplitudes ***z*** in a 3 × 3 × 3 neighbourhood *as seen from the centre of voxel* ***v***. By including all SH coefficients, the second spatial term affects the estimation of both even and odd components. Specifically, we create a conjunction fod, comprised of *M* = 252 points on the sphere (obtained by a sphere geodesic tessellation). For each point *i* (1≤*i*≤*M*) and respective orientation ***u***_***i***_, we define a value ***z***_*i*_ using the fods of neighbouring voxels (Fig. 2B).

One possible option for defining ***z*** could be to set $z_{i}=\mathrm{fo}d_{x}\left(-u_{i}\right)$ where the fod is taken from the voxel ***x*** in a neighbourhood of ***v*** such that the line connecting the centres of ***x*** and ***v*** is closest to ***u***_*i*_ (Fig. 2A). However, we instead use a soft version of the above to minimize the discretising effect of a voxel grid. We define ***z*** as a weighted sum over neighbouring fods:

(4) $$z_{i}=\frac{1}{c}\sum_{j\subset N\left(v\right)}e^{\frac{-\alpha_{j}}{\beta }}\mathrm{fo}d_{j}\left(-u_{i}\right)$$

where *α*_*j*_ is the angle between vectors ***u***_***i***_ and the line connecting the centre of the voxel ***v*** to the centre of a neighbour ***j*** belonging to a subset of a 3 × 3 × 3 neighbourhood of ***N(v)***. Specifically, a neighbour ***j*** is included in the estimation of the conjunction fod ***z*** if *α*_*j*_ < 90°. The parameter *β* can be empirically set and controls the slope of the exponential weighting function and *c* is a normalizing constant. Finally, and similar to the voxel-wise estimation, we enforce the positivity of the fod by adding the linear constraint $Bf_{\mathrm{all}}\geq 0$.We use a quadratic problem solver to minimize Eq. (3) iteratively until convergence. The iterative updates are needed as, contrary to a voxel-wise estimation, our scheme considers all data from a neighbourhood of voxels at the same time. Therefore, all neighbourhoods of a volume are swept through, before going to the next iteration. The fod continuity term in the cost function (Eq. (3)) is updated at the end of each iteration, when all the voxel-wise fods have changed. We initialize the optimisation by fitting voxel-wise symmetric fods in the first iteration using unconstrained spherical deconvolution (Tournier et al., 2004). These are then updated in subsequent iterations to reflect neighbourhood information.

### 2.2 Extension to multi-shell data using multi-tissue fibre responses

The estimation of A-fods can become problematic at tissue interfaces and might therefore require some extra constraints (Reisert and Kiselev, 2011). This is mainly because a single response function might not be enough to obtain fods representing actual fibre populations in isotropic regions such as grey matter and CSF (Roine et al., 2014). Recent work (Jeurissen et al., 2014) has shown that the classical non-parametric spherical deconvolution framework (Tournier et al., 2004, Tournier et al., 2007) can be extended to deal with multi-shell data (i.e., datasets acquired using multiple b-values). Moreover, including different response functions for three different tissue types (white matter, grey matter and CSF) improves the precision of fods. The same idea can be applied for A-fod estimation using the proposed approach. Equation (3) can be extended to:

(5) $$\hat{f_{\mathrm{all}}}=arg\underset{f_{\mathrm{all}}}{\min}\left\{{\left\|\left[\begin{array}{ccc} C_{1,1} & \cdots & C_{1,p}\\ \vdots & \ddots & \vdots \\ C_{k,1} & \cdots & C_{k,p} \end{array}\right]\left[\begin{array}{c} f_{\mathrm{even}}^{1}\\ \vdots \\ f_{\mathrm{even}}^{p} \end{array}\right]-\left[\begin{array}{c} Y_{1}\\ \vdots \\ Y_{k} \end{array}\right]\right\|}^{2}+\lambda^{2}{\left\|\left[\begin{array}{ccc} B_{1} & \cdots & 0\\ \vdots & \ddots & \vdots \\ 0 & \cdots & 0 \end{array}\right]f_{all}^{1}-z\right\|}^{2}\right\},\;with\;\left[\begin{array}{ccc} B_{1} & \cdots & 0\\ \vdots & \ddots & \vdots \\ 0 & \cdots & B_{p} \end{array}\right]\left[\begin{array}{c} f_{\mathrm{all}}^{1}\\ \vdots \\ f_{\mathrm{all}}^{p} \end{array}\right]\geq 0$$

where ***C***_*k,p*_ are matrices used to model the k-th shell and p-th tissue type, ***f***^*p*^ contains the SH coefficients of the A-fod for the p-th tissue type, ***Y***_*k*_ is the acquired dMRI signal in a voxel for the k-th shell and (***B***_*p*_***f***^*p*^) is the A-fod at ***v*** for the p-th tissue type. In the present work, we consider three main tissue types: white matter, grey matter and CSF. The first tissue type (*p* = 1) is white matter, and the fod continuity constraints only apply to this compartment.

Following this approach, we need to measure *k* x *p* response functions, i.e., one for each acquired b-shell and tissue type. Tissue types are derived using a T1 segmented volume brought to native dMRI data space. For each tissue type, 300 voxels were selected to obtain the response function. These voxels were randomly selected for grey matter and CSF and the maximum harmonic order was fixed at 0 (isotropic model). For the white matter response function, the 300 voxels within the white matter with the highest fractional anisotropy (and higher than 0.7) were selected. Their signal was then averaged to produce a fibre response function after aligning their principal diffusion direction as estimated from a diffusion tensor fit with the z-axis (Tournier et al., 2004).

### 2.3 Tractography using A-fods

Classical local tractography algorithms initialize a set of streamlines from every seed point and propagate them using orientations suggested by the local fods at each step. When a streamline enters a new voxel, the new direction of propagation is obtained by sampling the local fod (or extracting its maxima). Since the fod is symmetric, the samples are drawn from a sub-set of the fod lying within the cone of propagation that is built around the last incident direction. Such local stepping approach would not use the full potential of A-fods as it does not ensure fibre continuity as defined in the previous section. For instance, in a case where fibres converge in one direction and fan out in the opposite direction, the symmetric fod will always suggest fibre dispersion and will lead to fanning out in both directions (Jbabdi and Johansen-Berg, 2011).

To take advantage of the A-fods, we re-designed the classical local fibre-tracking approaches. Because of the sub-voxel information, it becomes crucial to take into account not only orientations and incoming propagation direction, but also the relationship between the streamline current location and the shape of the fod seen at this location. For instance, a streamline entering a voxel where fibres fan should select its next propagation orientation by also considering the fanning polarity. In practice, given the potential asymmetry in A-fods, we need to take into account not only the fod part that lies within the cone of propagation defined by the last stepping direction, but also the part on the opposite side. This would allow propagation that respects the fibre continuity assumption.

**Fig. 3:**
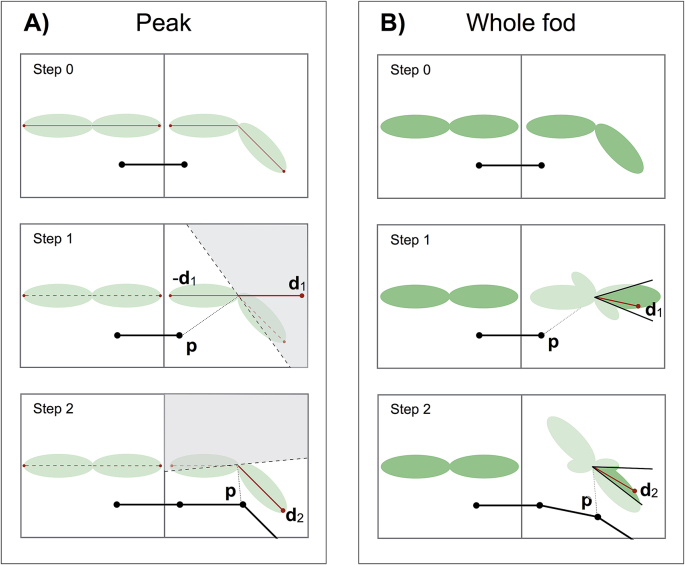
Peak-based and whole-fod-based tractography algorithm for a sharp sub-voxel bending fibre configuration. Step 0 represents the current streamline (black line) endpoints (black dots) overlaid onto two neighbouring A-fods. A) Step 0 of the peak-based case shows the peaks (red arrows) extracted from the underlying A-fods. At step 1 and step 2, the thicker red arrows represent the selected A-fod peaks (***d***_***1***_ and ***d***_*2*_ respectively) used for the next propagation steps. Such maxima were chosen as they lie within the sub-volumes (non-shaded area) identified by the current position vector ***p*** (dotted lines) and the plane perpendicular to it at the voxel centre (dashed lines). At each step, different non-relevant peaks (red dashed lines) are discarded. B) At step 1 of the whole-fod-based case, after A-fod weighting using ***γ***, direction ***d***_***1***_ lying within the user-defined cone of propagation (non-shaded area delimited by black lines) is selected using rejection sampling. At step 2 direction ***d***_***2***_ is chosen after weighting the A-fod using the same rationale.

We propose two variants of a tractography algorithm: one that uses fod peaks and one that samples from the whole fod. The former works as follows: peaks are pre-extracted from each fod using Powell's method (Jansons and Alexander, 2003). When a streamline enters a new voxel, a virtual plane is defined. Such plane passes through the centre of the voxel and is perpendicular to the position vector ***p*** pointing from the centre of the voxel to the current position of the streamline (Fig. 3A). This plane divides the voxel into two parts and it is used to select the peaks of the A-fod that should be used for the next direction of propagation. The algorithm discards all fod peaks that do not lie on the same side of the plane as the streamline current location ***p***. Note that these steps would leave peaks of symmetric fods unchanged. The remaining peaks define potential orientations for propagation. The algorithm then proceeds as a conventional streamline tractography: a new cone of propagation is built around the last direction of propagation ***d***_*j*_; from the remaining peaks the one that lies within the cone of propagation and is most collinear to the last incident direction ***d***_*j*_ is selected (Fig. 3A) and a step is made along that peak orientation.

In the case where the full fod is used (rather than just its peaks), we use a different approach. The A-fod at the current voxel and location is weighted as a function of the position vector ***p*** relative to the fod:

(6) $${fod^{\prime }\left(u_{i}\right)|}_{p}=\gamma \left(p,u_{i}\right)\mathrm{fod}\left(u_{i}\right)+\left(1-\gamma \left(p,u_{i}\right)\right)\mathrm{fod}\left(-u_{i}\right)$$

where $\mathrm{fod}\left(u_{i}\right)$ is the A-fod evaluated along direction ***u***_*i*_ and:

(7) $$\gamma \left(p,u_{i}\right)=\{\begin{array}{l} 0\\ p\cdot u_{i}+0.5\\ 1 \end{array}\begin{array}{c} \;\;\;if\;p\cdot u_{i}<-0.5\\ \;\;\;\;\;\;\;\;\;\;\;\;\;\;\;\;if-0.5<p\cdot u_{i}<0.5\;\\ if\;p\cdot u_{i}>0.5 \end{array}$$

This weighting step promotes the relevant lobe of the A-fod relative to the current streamline position within the current voxel (Fig. 3B) and uses the opposite of this lobe to provide a symmetric estimate. Again, notice that symmetric fods are left unchanged by this step. The function γ is a smooth version of the step-function used in the first algorithm in the form of a plane. A key feature of this approach is that in the case of fibre fanning, this algorithm forces streamlines to take into account fanning polarity such that groups of streamlines converge or diverge depending on their incoming directions. After the location-dependent weighting of the fod, rejection sampling is used to sample candidate orientations within the cone of propagation built around the last stepping direction.

### 2.4 Quantifying asymmetry

In order to quantify the degree of asymmetry in the orientation distributions fitted by our model, we use the fod power. Since the spherical harmonics basis is orthonormal, overall power can be calculated as the sum-of-squares of the coefficients:

(8) $$P=\int_{S}{\left|\mathrm{fod}\left(u\right)\right|}^{2}du=\sum_{l=1}^{L}\frac{1}{2l+1}\sum_{m=-l}^{l}{\left|f_{lm}\right|}^{2}$$

where *P* is the power of the fod, *L* is the maximum harmonic order, and ***f***_*lm*_ the spherical harmonic coefficient of order *l* and degree *m*. To compute the power of the asymmetric and symmetric fod components, we use the odd and even order coefficients in the above sum respectively.

## 3 Methods

### 3.1 Histology-based simulations

To assess the ability to estimate sub-voxel information using A-fods, we extracted fibre patterns from high-resolution histology of a macaque brain. We used image processing to estimate fibre orientations at ∼μm scale and used these orientations to simulate dMRI signal. We then downsampled the signal at MR resolution levels (∼mm scale) and applied single-shell and single-tissue A-fod estimation. Finally, we compared the estimated sub-voxel patterns (from the low-resolution data) with the ground-truth high-resolution (from histology).

More specifically, a postnatal day 6 macaque brain was perfusion-fixed with 4% paraformaldehyde and postfixed for 24 h at 4 °C temperature. The sample was then sectioned coronally at a slice thickness of 70 μm using a Vibratome. Slices were immunostained with antibody to myelin basic protein (MBP, MAB395, Millipore) at 1:100 dilution and using Vectastain (Vector, USA) immunodetection kit with VIP (Vector) as chromogen. After mounting, dehydration and coverslipping, sections were scanned using a NanoZoomer 2 (Hamamatsu) microscope equipped with an Olympus lens (the final in-plane resolution of the images was 50 μm × 50 μm).

Histological sections were processed using pixel-wise structure tensor analysis (Budde and Frank, 2012, Sotiropoulos et al., 2013a). Intensity gradients were calculated on grayscale images, after smoothing using a 2D Gaussian kernel (to increase stability in gradient calculation). A structure tensor was then obtained for each pixel, using the gradients of the pixel's local neighbourhood. The eigenvector of the structure tensor corresponding to the smallest eigenvalue gave the coherence direction, i.e. the direction along which the image intensity exhibits the lowest fluctuations (Weickert, 1999). These pixel-wise directions provide a good description of the underlying neuronal fibre orientations, as traced by the histological staining process.

The diffusion MRI signal was simulated using the structure tensor estimates and the ball and stick model (Behrens et al., 2003). The orientation extracted from the structure tensor and a diffusivity of 0.7 μm^2^/ms were used. The fractional volume *f* of each stick was obtained using the grey level index (GLI) of the stained section's image using $f=1-\frac{GLI}{255}$. The b-value was set to 2000 s/mm^2^. Once the signals were simulated at the high-resolution of histology, they were downsampled to 1 mm × 1 mm pixel sizes. This was performed by summing signal contributions arising from every element of a 20 × 20 pixel grid. Rician noise (σ = 0.05) was then added to the simulated signal to obtain an SNR of 20 (i.e., S_0_/σ, where S_0_ is the amplitude of the b = 0 signal – S_0_ = 1 was assumed in the simulations). A-fods were then estimated using the proposed approach (and a 3 × 3 neighbourhood) and S-fods using the approach proposed by (Tournier et al., 2007).

### 3.2 Histology-based A-fod validation

Histology-extracted fods (Hist-fods) that reflect sub-voxel fibre patterns were used to evaluate the results of the proposed A-fod estimation framework. For each downsampled pixel, Hist-fods were computed by reconstructing angular histograms of the orientations obtained from structure tensor analysis at high-resolution and were contained within the respective pixel. Each histogram comprised 360 bins (=1° angular resolution). To allow asymmetry representation in the estimated histograms, each bin quantified the number of orientations that were oriented along the same direction and that lay within the sub-volume identified by the voxel's boundaries and the plane perpendicular (at the voxel centre) to the current sampled direction. The angular histogram was then smoothed by convolving it with a Gaussian window of 23° full-width at half maximum (FWHM). This value was obtained using a non-parametric normal kernel density estimation approach and represents the average bandwidth of the kernel smoothing window (Seehaus et al., 2015). Then, the correlation between Hist-fods and the estimated fods was computed in each down-sampled pixel (i.e., 1 mm isotropic resolution).

### 3.3 Histology-based tractography

We further evaluated the performance of the proposed tractography approaches. When using the peak-tracking approach, a maximum number of 3 (6) peaks were extracted from the S-fod (A-fod) field using Powell's method (Jansons and Alexander, 2003). Euler's integration method was then applied on the trilinearly interpolated vector field of fod peaks. To explore benefits, we compared results obtained using A-fod-based tractography to the downsampled data with results obtained by applying 1) conventional streamline tractography to the very high-resolution orientations estimated using structure tensor analysis and 2) S-fod-based tractography with trilinear interpolation to the downsampled data. Our histology-based simulations were two-dimensional, therefore the tractography examples were limited to lie within the same plane. Step-size, angular threshold, and fod threshold were set to 20% of the pixel size, 40° and 0.1 respectively.

**Fig. 4:**
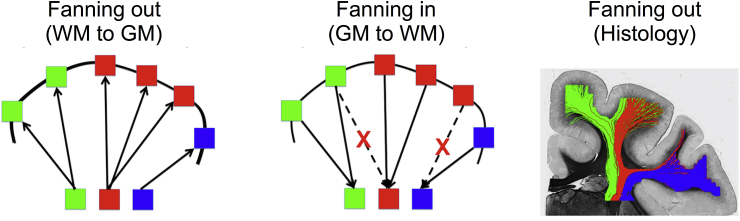
Cartoon representation of the fanning in and out configurations (left and middle panels). Streamlines are colour-coded based on their seed ROI of origin and overlaid on top of a coronal myelin-stained section (right panel).

To further assess the potential of using A-fods in combination with the proposed tractography approach, a topography-preservation experiment was performed. The idea was to test whether topographical organisation obtained when tracking a *spreading* pattern (WM to cortex) is preserved when tracking in the opposite *converging* direction. A region of interest (ROI) was defined comprising the white matter dorsal and lateral to the putamen as it enters the internal capsule. From each ROI pixel, 5000 streamlines were seeded once using S-fods and once using the proposed A-fods (fanning out, Fig. 4). Grey matter pixels that were reached by at least one streamline were grouped together to form a binary mask (GMHitMask). The initial seed ROI was then sub-divided into three smaller ROIs (Fig. 4). A number was assigned to each grey matter voxel belonging to GMHitMask, identifying the sub-ROI that was mostly connected to it. Lastly, 5000 streamlines were seeded from each grey matter pixel within GMHitMask (fanning in, Fig. 4). The fraction of streamlines that correctly reached the initial seed WM ROIs was then quantified to assess whether A-fods help preserving the topography of these connections.

### 3.4 MRI data analysis

Diffusion MRI and T1-weighted data were obtained from the WU-Minn Human Connectome Project (HCP) (Sotiropoulos et al., 2013b, Van Essen and Ugurbil, 2012). Briefly, the HCP dMRI protocol uses a single-shot 2D EPI acquisition with Stejskal-Tanner pulsed gradients (Multiband factor = 3, 1.25 mm^3^ isotropic resolution, 110 slices, partial Fourier = 6/8, 3 b-value shells = 1000-2000-3000 s/mm^2^, 90 dMRI volumes for each shell + 5b0s acquired twice varying the phase encoding direction, TE/TR = 89/5500 ms). Standard minimal pre-processing was run on the data to correct for eddy current and susceptibility induced distortions, bulk motion and gradient non-linearity (Andersson and Sotiropoulos, 2016, Glasser et al., 2013, Sotiropoulos et al., 2013b).

A-fods and S-fods were fitted to the multi-shell data using the multi-shell multi-tissue approach and a maximum harmonic order of 8 in white matter voxels in 10 different HCP subjects (3 × 3 × 3 neighbourhoods used for the A-fods). Maps of (a)symmetric coefficients were estimated for each individual subject and averaged in MNI space to identify white matter areas where fods are more asymmetric. Similar to the histology data analysis, tractography experiments were performed to assess the benefits of the proposed approach in estimating topographic organisations.

A topography-preserving experiment was performed to assess the benefit of resolving fanning polarities. Pathways spreading from the internal capsule to the cortex and converging from the cortex to the internal capsule are good candidates for such an experiment. An ROI in standard MNI space of the left internal capsule (IC) was obtained using the ICBM atlas (Mori et al., 2008, Mori and van Zijl, 2007, Wakana et al., 2004) (by grouping together the anterior and posterior IC parts at axial slice z = 64). The ROI was nonlinearly warped to the diffusion space of 10 HCP subjects (Andersson et al., 2010). First, 5000 streamlines were propagated from each IC voxel and only those reaching the white-grey matter boundary were retained. Each IC voxel was assigned a label *L* (see labels in Fig. 11) representing the cortical area (Desikan et al., 2006) at which streamlines (seeded from that voxel) mostly terminated. Then, the experiment was repeated in the opposite direction; 5000 streamlines were seeded from the WM/GM boundary vertices reached in the previous step. Only those streamlines reaching the original IC mask were retained. Tractography was performed using both the symmetric and asymmetric whole-fods and peak-based tractography (step size and curvature threshold were identical). To assess topography preservation, streamlines from each cortical region *A* to all labels *L* in the IC (i.e., the ensemble of IC voxels that preferentially projected to cortical region L) were counted. Preserved topography requires that the count *A-A* is higher than any other count *A-L* with *L*≠ *A*.

Finally, in the absence of ground-truth for the in-vivo data, we assessed the agreement of the connection patterns estimated using S-fod and A-fod tractography with connectivity estimated using a different MRI modality (resting-state functional MRI).

### 3.5 Hyper-parameter selection

The A-fod cost function (Eqs. (3), (5)) has a number of hyper-parameters that we need to set for the optimisation. These include the regularization parameter *λ* and the number of iterations. We used a k-fold cross-validation to find the optimal *λ*. Thirty thousand random white matter voxels from an HCP dMRI dataset and 61 candidate values for *λ*, logarithmically spanning the interval between 0.01 and 10, were considered. The signal in each of these voxels was divided into *k* partitions of *n* signal values and the values from *k-1* sets were used to fit the proposed model. To test the goodness of fit on the *k-*th partition we computed the mean squared error (MSE) between the predicted and the measured signal. The *λ* parameter that was minimizing the MSE in every voxel was selected. This operation was repeated 61 × *k* times by considering a different signal partition at each iteration. More specifically, we set *k* to 10, for a total of *n* = 9 unique samples for each partition. We found that the optimal regularization parameter was *λ* = 0.1 (this was the mode of the distribution of optimal *λ* across all voxels).

Convergence of the A-fod estimation procedure was evaluated by computing the average sum of squared differences of the spherical harmonics coefficients across the whole data volume between the current iteration and the previous one. A threshold was empirically set after visual inspection of simulated and in vivo data to 0.001. On average, the algorithm converged in 3 iterations. The angle *β* that determines the slope of the weighting function when calculating vector ***z*** (Eq. (4)) was set to 40° to limit the influence of neighbouring directions that might smooth fod peaks (Ehricke et al., 2011).

## 4 Results

### 4.1 Histology-based simulations

**Fig. 5:**
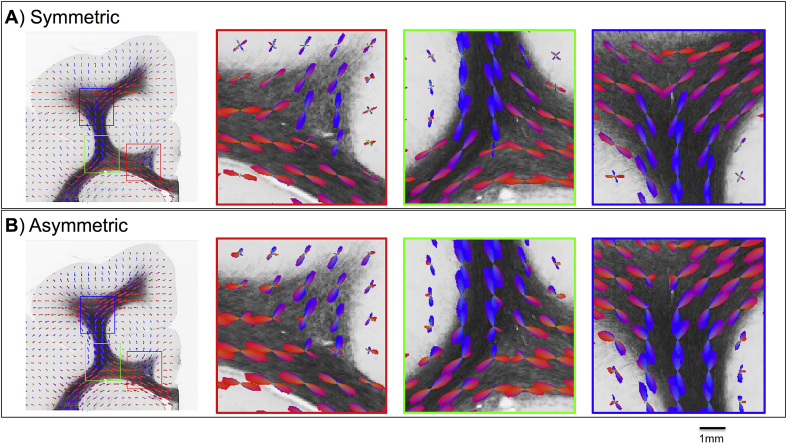
Comparison of A) symmetric and B) asymmetric fods estimated on simulated data with fibre patterns extracted from histology. Fods are overlaid on myelin-stained section. The three zoomed insets show how A-fods are capable of better delineating three-way splitting (first & second column) and fibres bending sharply to enter cortical grey matter (third column). Fods are colour-coded based on a 2D colour map (red for left-right and blue for up-down).

Fig. 5 shows examples of S-fods and A-fods from simulated data with the underlying fibre patterns extracted from myelin stained sections. The A-fods depict Y-shaped fibre splitting, C-shaped bending as well as fanning polarity, where S-fods estimate crossings or symmetric orientation dispersion.

**Fig. 6:**
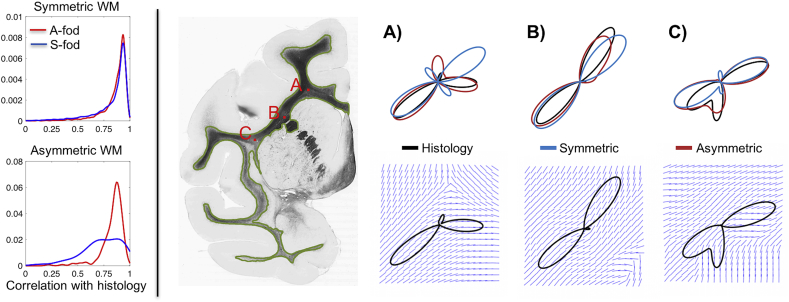
Comparison between histology-based, symmetric and asymmetric fods. Left panel shows histograms of correlations between fods and Hist-fods across ten different histological sections. A distinction is made between fibre patterns that exhibit high or low asymmetry (i.e., where the power of the asymmetric component of the A-fod is higher or lower than 0.01). The right panel shows some example fods from a sample histological slice shown on the left (white/grey matter boundary overlaid in green). A) non-symmetric fibre crossing/splitting, B) fibre fanning & bending and C) fibre bending. Polar plots show the three different fods on top of each other colour-coded according to the legend at the bottom. The bottom row shows the three histology-based fods overlaid onto the high resolution structure tensors comprised within the simulated 1 mm isotropic voxel.

To quantify these differences, we compared the fibre patterns suggested by A-fods and S-fods (estimated at low resolution - mm scale) to “ground-truth” histology-based fods (Hist-fods) (computed at the high resolution – μm scale). As shown in Fig. 6, A-fods are matching Hist-fods better than S-fods. Particularly in voxels with asymmetric patterns, A-fods correlate significantly better with Hist-fods compared to S-fods. In such voxels, the median correlation with Hist-fods was equal to 0.73 and 0.86 when using S-fods and A-fods respectively. In voxels with symmetric patterns, the benefit of using A-fods is smaller; the median correlation with Hist-fods was equal to 0.89 and 0.9 when using S-fods and A-fods respectively. The examples shown in the Figure are derived from three different voxels, with the ground-truth patterns reflecting a combination of fibres splitting, fanning and bending. The correlations between the angular histograms of S-fods and Hist-fods were: A) 0.4, B) 0.78 and C) 0.84. When using A-fods, the correlations improved significantly: A) 0.87, B) 0.9 and C) 0.95.

**Fig. 7:**
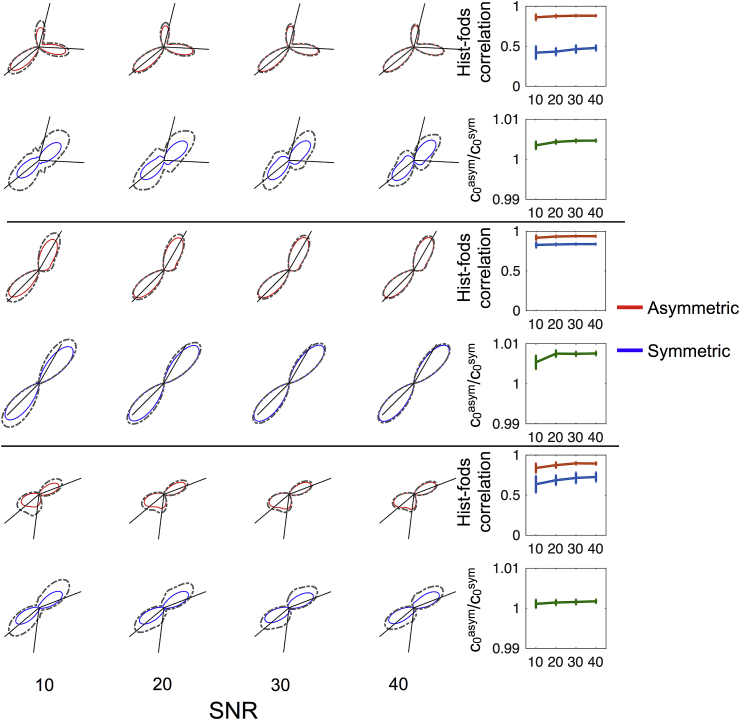
Simulation results for the three voxels shown in Fig. 6. Mean A-fods (S-fods) over 50 repetitions and at 4 different SNRs are shown as red (blue) outlines. Mean amplitudes + 2 standard deviations are shown as dashed grey contours. Black lines represent peaks extracted from the corresponding ground truth Hist-fods. Plots in the rightmost column show the average correlations (error-bars = 1 standard deviation) between Hist-fods and A-fods (S-fods) and average ratios (error-bars = 1 standard deviation) between the 0-th order asymmetric and symmetric harmonic coefficients.

We assessed the accuracy and precision of the proposed approach by re-running the fod estimation on 50 different noise realizations of each MRI-simulated voxel from a single histological section at 4 different SNR levels. Fig. 7 focuses on the same voxels presented in Fig. 6 and shows that our proposed approach is quite robust even when SNR is low (∼= 10). Moreover, as amplitudes are consistent between asymmetric and symmetric fods, the proposed A-fod estimation routine does not affect apparent fibre densities for corresponding peaks. This is also confirmed by the ratios between the 0-th order spherical harmonic coefficients (used here as proxy for average fibre density) of A-fods and S-fods that is consistently close to 1.

**Fig. 8:**
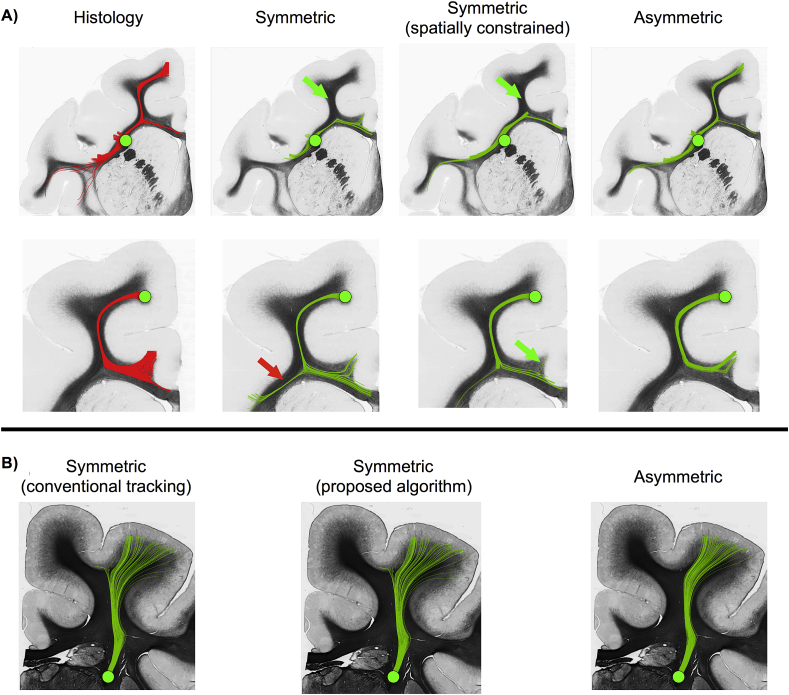
A) Peak-based tractography results using two seeds and four different vector fields (histology-based orientations at μm scale, peaks extracted from the symmetric, spatially constrained symmetric and asymmetric fods at mm scale). Upper row shows the streamlines obtained when seeding from deep white matter. Bottom row shows the streamlines obtained when seeding from the WM/GM boundary. Asymmetric fod-based tractography limits the number of false negatives (green arrow) and false positives (red arrows) when compared to the ground truth obtained using histology. B) Peak-based tractography results using one seed and two different vector fields (peaks extracted from the symmetric and asymmetric fods at mm scale). First panel shows results obtained when using classical symmetric tractography. Last two panels show results obtained using the proposed asymmetric tractography algorithm on the two different vector fields.

We further tested how well we can utilise these local estimates in the proposed A-fod-based tractography algorithm. Conventional peak-based tractography at μm resolution using the histology-extracted orientations was considered to give ground-truth trajectories. The proposed tractography method was applied to A-fods estimated at mm resolution, while conventional tractography (Euler integration with trilinear interpolation) was applied to S-fods and spatially constrained S-fods (i.e., the second term of Eq. (3) now minimizes the difference between the symmetric fod (***Bf***_*even*_) and the conjunction fod ***z***). Fig. 8A shows results for two different seed points. In both cases, A-fod-based tractography reduces the number of false turns and follows more accurately the histology-derived trajectories. Importantly, when then underlying fibre patterns are not particularly asymmetric, the results look very similar when using peaks extracted either from S-fods or A-fods (Fig. 8B). Moreover, applying the A-fod-based tractography strategy does not change the results when tracking through a S-fod-based vector field. These results suggest that the proposed algorithm improves tracking when asymmetric patterns are present, but does not have detrimental effects in the absence of such patterns.

**Fig. 9:**
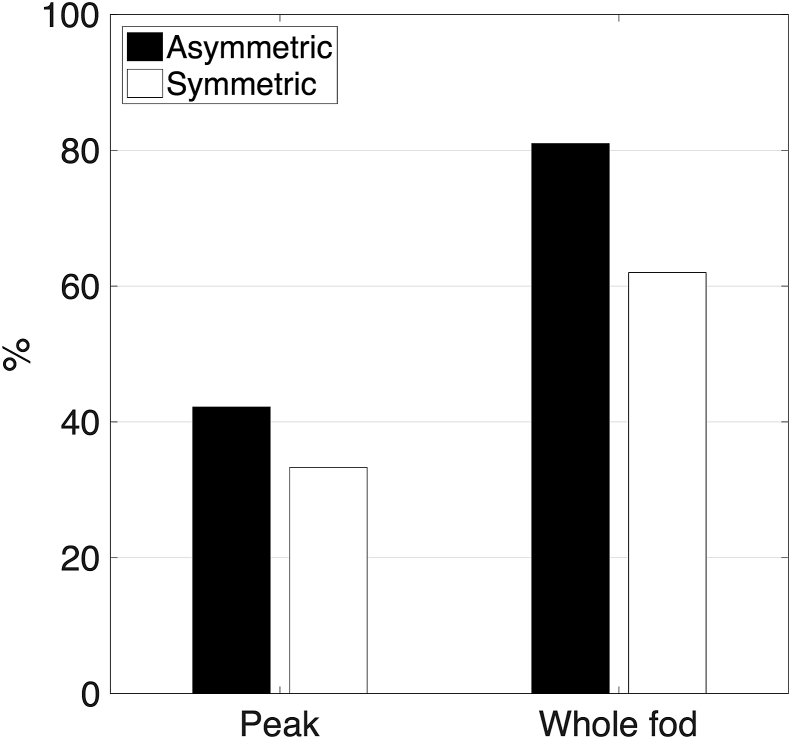
Results of the topography preservation experiment (Fig. 4) performed using symmetric and asymmetric fods. The vertical axis represents the percentage of correct back-projections (i.e. percentage of deep WM locations that projected preferentially to certain WM/GM boundary locations and were subsequently reached back by projections from these WM/GM boundary locations). Asymmetric fods-based tractography gives consistently better results using both a peak-based (left) and a whole-fod-based (right) tractography approach. This is shown by a higher percentage of correct back-projections.

Finally, we explored the potential to resolve correctly and use fanning polarity. We performed a topography-preservation experiment (see Fig. 4 and relevant section in Methods) to check how well organisation revealed through a spreading fibre pattern is preserved after tracking in the opposite direction, where trajectories are expected to converge back to the same topography. Fig. 9 shows the results of the experiment for both tractography approaches. Topography is better preserved when using an A-fod-based tractography. In the peak-tracking case, 9% more WM/GM boundary voxels projected streamlines back to the original seed ROI, when using A-fods compared to S-fods. In the whole-fod case, 19% more WM/GM boundary voxels projected back to the original seed ROI. The reduction of false positives using A-fods suggests that the correct fanning polarity is better captured.

### 4.2 In vivo results

**Fig. 10:**
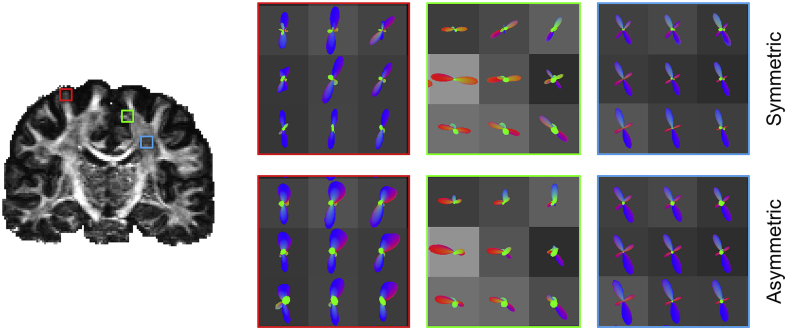
Comparison between in symmetric (top row) and asymmetric fods (bottom row) overlaid on a coronal FA map (subject ID = 100408). The zoomed insets show 3 different ROIs where fibres fan (red inset), bend sharply (green inset) or cross (blue inset).

Fig. 10 shows examples that qualitatively illustrate differences between A-fods and S-fods estimated using in-vivo HCP data. A-fods seem to capture complexity in cases where we would anticipate fibre pattern asymmetry. For instance, they represent fibre fanning and polarity at the gyral crown (red inset) and depict sharp bends and fans in the case of complex fibre configurations (green inset). Moreover, they preserve fibre crossings in regions where these are expected, such as in the centrum semiovale (Fig. 9, blue inset).

**Fig. 11:**
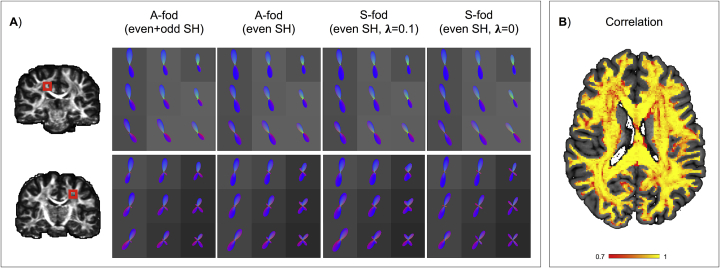
In vivo regularization of the symmetric component. A) The ROI shown in each row is highlighted using a red square overlaid on the subject's FA map. Even order SH coefficients are extracted from the original A-fods to obtain S-fods. These visually match the S-fods obtained by performing symmetric CSD, meaning that the optimal fit to the signal is preserved after a small amount of spatial regularization. Full A-fod and its even component show sharper and better defined peaks when compared to the spatially constrained S-fod. B) Correlation map of fod amplitudes (sampled from 252 evenly distributed points on a sphere) obtained from S-fods and symmetrised A-fods for a single subject (ID = 100408) overlaid on top of the subject's T1-weighted acquisition.

To explore the effect of the neighbourhood term in the estimation, Fig. 11A shows a comparison between the estimated A-fods, the A-fods comprising only of even order spherical harmonics and the S-fods obtained when setting λ=0.1 (i.e., performing spatially constrained symmetric-only spherical deconvolution) and λ=0 (i.e., performing traditional symmetric-only spherical deconvolution without any spatial information) in two white matter ROIs. Considering only the even SH coefficients makes the asymmetric estimate (i.e. with the neighbourhood term) look very similar – but not identical – to the voxel-wise symmetric estimate. This shows that there is a small amount of spatial regularization in the A-fods, however the optimal fit to the voxel-wise signal is preserved (Fig. 11A). Moreover, applying a spatial constraint to S-fods seems to affect the angular resolution of the reconstructed fods. To quantify the comparison, we computed the correlation between the amplitudes of S-fods and the symmetric (i.e., even order SH) components of A-fods. Considering all white matter voxels of 10 HCP subjects the average correlation was 0.91 (median = 0.96, std = 0.14), confirming that the two are very similar (Fig. 11B). The neighbourhood term does induce a soft spatial regularization in the even components, however the main differences come from the incorporation of the odd SH components and their estimation through this term (leftmost column in Fig. 11A).

**Fig. 12:**
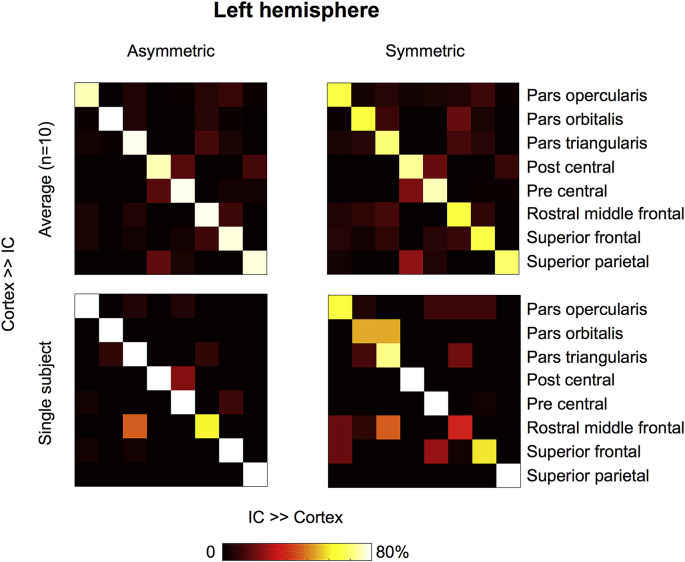
Results of the topography preservation experiment performed on in vivo data using a local whole-fod tractography approach. The matrices show the row-normalised average streamline counts that project back to a patch in the IC, which was preferentially projecting to the same cortical area. Top row shows the results averaged across 10 HCP subjects (IDs: 100307, 100408, 101915, 102816, 103414, 103515, 103818, 105115, 105216, 106016), bottom row shows the results for a single HCP subject (ID = 100408). The mean of the single subject diagonal values for the asymmetric case is 86.3% and 71.3% for the symmetric case. Using the proposed asymmetric fods-based tractography algorithm, more streamlines project back to their original cluster within the internal capsule. This is shown by higher values on the matrix diagonal.

Fig. 12 shows the results of the in vivo topography-preserving experiment in the left hemisphere for the group (n = 10 subjects) and for a single HCP subject (ID = 100408) using the whole-fod tractography approach. Topography was found to be better preserved when using A-fod-based tractography. More WM/GM boundary vertices projected back to the original seed ROI when considering asymmetry. The mean of the diagonal values averaged across 10 different subjects obtained using A-fods (S-fods) whole-fod-based tractography is 77% (67%) in the left hemisphere and 77% (68%) in the right hemisphere (Supplementary Fig. S1). The average standard deviation of the diagonal elements obtained using A-fods (S-fods) whole-fod-based tractography is 9.7% (11.1%) in the left hemisphere and 10.2% (12.0%) in the right hemisphere. The mean of the diagonal values averaged across 10 different subjects obtained using A-fods (S-fods) peak-based tractography is 49% (40%) in the left hemisphere and 43% (40%) in the right hemisphere. The average standard deviation of the diagonal elements obtained using A-fods (S-fods) peak-based tractography is 23.6% (18.6%) in the left hemisphere and 23.7% (20.0%) in the right hemisphere.

**Fig. 13:**
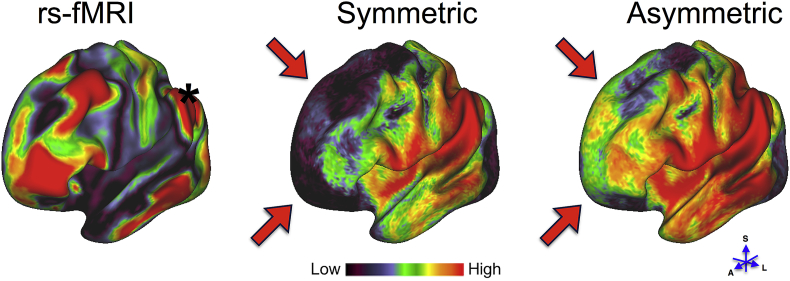
Seed-based functional (first panel) and structural (second and third panels) connectivity maps averaged over 10 subjects (IDs: 100307, 100408, 101915, 102816, 103414, 103515, 103818, 105115, 105216, 106016) and overlaid on an inflated left hemisphere surface. Black asterisk identifies the TPJ seed region. Red arrows point at cortical areas with obvious qualitative differences in connectivity strength between symmetric and asymmetric whole-fod-based tractography. To aid visualization, the functional connectivity map shows the absolute value of the fMRI time-series correlation.

In the absence of ground-truth for the in-vivo data, we used similarity of tractography predictions with results from a different modality to indirectly evaluate the accuracy of results. Specifically, we performed a seed-based connectivity analysis to test whether using A-fod-based tractography improves the structural and functional connectivity profile similarity. Two regions comprising the left and right temporoparietal junction (TPJ) were manually drawn on the cortical surface in MNI space. 5000 streamlines were seeded from each surface vertex within TPJ. Streamlines were stopped when they reached the ipsilateral pial surface or the mid-sagittal plane and counted when they crossed the WM/GM boundary. These streamline counts across the whole WM/GM boundary surface were obtained for 10 HCP subjects and the resulting matrices were averaged after normalizing each of them by the total number of streamlines that successfully reached the WM/GM boundary for each subject. Functional connectivity matrices were also computed using resting state fMRI data from the HCP (Smith et al., 2013). The correlation between the average fMRI time-series of the TPJ with the time-series at each cortical surface vertex was used to obtain a functional connectivity map. The similarity between the functional and structural connectivity profiles of the TPJ was compared and found to be improved when using A-fod-based whole-fod tractography. Qualitatively, Fig. 13 shows that A-fod based whole-fod tractography better matches the functional connectivity strength between the left TPJ and the medial aspects of the frontal lobe (Supplementary Fig. S2 shows the results obtained when seeding from the right TPJ). To quantify the similarities, we computed the *partial* correlation between the functional connectivity and the structural connectivity profiles obtained using A-fod whole-fod-based tractography, after regressing out the S-fod tractography results (r_left_ = 0.30, r_right_ = 0.26). Similarly, we computed the partial correlation between the functional connectivity and S-fod-based tractography results, after regressing out the A-fod tractography results (r_left_ = −0.26, r_right_ = −0.22). These results illustrate that, when not taking into consideration the information shared between S-fod and A-fod results, A-fod tractography adds information that improves agreement with fMRI-based predictions, while S-fod tractography does not.

**Fig. 14:**
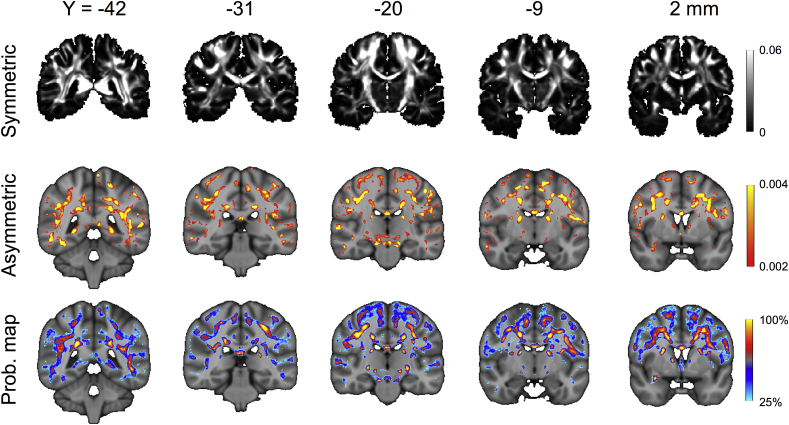
Power of the symmetric (top row) and asymmetric (middle row) component of the A-fods. The bottom row shows probability of asymmetry occurrence maps obtained by pooling results from 10 subjects (IDs: 100307, 100408, 101915, 102816, 103414, 103515, 103818, 105115, 105216, 106016). The map illustrates voxels that consistently show an asymmetric coefficient value higher than 0.002 in more than 25% of the subjects. Both the asymmetric power and the probability maps are overlaid on the T1 MNI template (1 mm isotropic, coronal views).

Finally, Fig. 14 shows the power of even and odd order SH coefficients for the A-fods estimated in the brain. The symmetry maps (top row, power of even order coefficients) have a very good CNR when trying to distinguish between white and grey matter and highlight areas where fibres are extremely symmetrically organised (e.g. ventral corticospinal tract). On the other hand, the asymmetry maps (middle row, power of odd order coefficients) reveal areas where sub-voxel fibre patterns are more complex than in the rest of deep white matter. Some examples include the dorsolateral part of the thalamus, the bottom of the body of the corpus callosum and regions peripheral to the centrum semiovale, where fibres head towards cortex. The locations of such areas with complex sub-voxel fibre configurations are consistent across individuals (bottom row, probability maps of the asymmetric coefficient).

## 5 Discussion

In the present work we introduced a new comprehensive framework to estimate asymmetric fods and track through complex geometries using dMRI data. The framework extends classical non-parametric CSD-based approaches by using information from each voxel's neighbourhood and imposes a fibre continuity constraint to resolve asymmetric fibre patterns. By using realistic fibre configurations as derived from high-resolution histological data, we show that the added asymmetric information can resolve realistic sub-voxel fibre configurations (e.g., fanning and bending) and that it improves tractography.

Traditional local stepping tractography approaches do not necessarily ensure fibre continuity. Furthermore, symmetric fods will always suggest fibre dispersion in cases where fibres converge in one direction and fan out in the opposite direction, leading to fanning out in both directions (Jbabdi and Johansen-Berg, 2011). Therefore, to respect the fibre continuity assumption, it becomes crucial to take into account not only orientations and incoming propagation direction of a streamline, but also where the streamline lies within a voxel relative to the fod. We proposed two new tractography solutions to this problem, one for each local tracking strategy. Both algorithms aim to ensure that the propagation directions reflect sub-voxel orientation information given the current streamline endpoint.

Using realistic complex fibre patterns derived from histology we have shown that A-fods obtained using the proposed approach are capable of improving the representation of sharp bends as well as fanning polarity. Moreover, the proposed approach improved the fod estimation also in simulated voxels classified as being purely symmetric. This is because no simulated noisy sub-voxel pattern is perfectly symmetric and the imposed threshold to discriminate between asymmetric and symmetric voxels does not ensure a perfectly binary distinction. The added information, together with the proposed tractography approaches, is shown to be valuable in yielding better reconstruction of connection's topographic organisation. These topographies are highly relevant when studying brain function as they reflect the ordered spatial organisation/segregation of sensory information and complex cognitive processes (Jbabdi et al., 2013).

We also showed that A-fods representing asymmetric fibre fanning and sharp bends are present in-vivo. Importantly, A-fods preserve fibre crossings in regions where asymmetric configurations are less likely, such as in the centrum semiovale. Areas where asymmetry is particularly pronounced seem to be very consistent across subjects (Fig. 14, bottom row). This might hint to a potential use of this measure as a marker in disorders where tract lateralization is compromised, but it is a topic that goes beyond the scope of the present work. Similar to the results obtained from histology using simulated realistic patterns, topographic properties are better preserved also in-vivo, when using A-fod-based tractography. Because of the lack of a proper in vivo gold standard for structural connectivity, we used a multi-modal approach to test whether structural connections from the TPJ as estimated using A-fod-based tractography better reflect its functional connections. Previous studies have shown that the TPJ is structurally and functionally connected to vast portions of the antero-medial and ventral prefrontal cortex (Mars et al., 2012). Our rs-fMRI results confirm this pattern, but we have found a clear qualitative and quantitative difference when looking at the structural connectivity indices obtained using A- or S-fods. Asymmetric-fod-based tractography results show an increase in seed-based structural connectivity to the medial prefrontal cortex when compared to S-fod-based results. A further partial correlation analysis shows that such increase in connectional strength is correlated to the functional connectivity profile. This might be hinting to the fact that, because of its capability to select only the appropriate fod peaks, streamlines sampled using A-fod-based tractography have a better chance to correctly reach distant cortical areas.

Previous approaches have been proposed to recover complex sub-voxel fibre patterns using post-processing regularization techniques (Barmpoutis et al., 2008, Ehricke et al., 2011), curve inference labelling (Campbell et al., 2014, Savadjiev et al., 2006) and tractography (Rowe et al., 2013). One notable approach infers A-fods directly from the data by imposing a geometric fibre continuity constraint (Reisert et al., 2012). Our framework uses an asymmetric basis set to describe A-fods, which is conveniently offered by the full (i.e., even and odd order) spherical harmonics basis (i.e., the results do not depend on the discrete sampling of the sphere) and distinguishes between tissue boundaries. Moreover, we have thoroughly validated our method using realistic fibre configurations and have shown clear benefits for tractography when explicitly taking into account the asymmetry information, which is non-trivial and core in our framework.

One limiting factor of the proposed method is that the width and amplitude (i.e., apparent fibre density) of the fod will be influenced by the chosen response function. Currently, the response function for each tissue type and for each b-shell is the same across the whole volume. If the response function itself captures a lot of fibre dispersion, the fods in each voxel will look sharper and vice-versa (Parker et al., 2013). This is a limitation shared with the traditional voxel-wise CSD approaches and certain improvements can be explored (Roine et al., 2015, Tax et al., 2014). Furthermore, to test whether our framework affects local apparent fibre densities (Raffelt et al., 2012), we looked at the difference between the 0^th^–order spherical harmonic coefficient (as a probe for apparent fibre density) of A-fods and S-fods. We found that, across white matter, the correlation between the 0^th^-order coefficients of A-fods and S-fods is equal to 0.997. We have also computed the median relative difference ($\left|\frac{{\text{c}}_{0}^{\text{asym}}-{\text{c}}_{0}^{\text{sym}}}{0.5\text{*}\left({\text{c}}_{0}^{\text{asym}}-{\text{c}}_{0}^{\text{sym}}\right)}\right|$) within white matter and found that to be equal to 0.0066. This indicates that A-fods share a similar behaviour and limitations with S-fods in representing apparent fibre densities.

Another limitation of the proposed tractography algorithm is the necessity to have a rather conservative angular constraint. This is done to prevent streamlines to take sharp and anatomically inaccurate turns in deep white matter regions (Bastiani et al., 2012). At the interface between white and grey matter, though, white matter fibres change their orientations abruptly (Cottaar et al., 2016, Sotiropoulos et al., 2013a). Therefore, limiting the angle at each propagation step might prevent to correctly reconstruct such sharp transitions. This could potentially be solved by implementing a global tractography approach based on A-fods that would allow us to relax the angular constraint and let fibres reaching gyral walls to sharply bend at the interface between white and grey matter (Reveley et al., 2015).
