# Supplementary material for: Improved tractography using asymmetric fibre orientation distributions
Source: Neuroimage. 2017 Sep;158:205–18. doi: 10.1016/j.neuroimage.2017.06.050 (PMC6318223; doi:10.1016/j.neuroimage.2017.06.050)
Supplement: Supplementary file 1 [file mmc1.docx]

Improved tractography using asymmetric fibre orientation distributions

Matteo Bastiani^1^, Michiel Cottaar^1^, Krikor Dikranian^3^, Aurobrata Ghosh^2^, Hui Zhang^2^, Daniel C. Alexander^2^, Timothy E. Behrens^1^, Saad Jbabdi^1^, Stamatios N. Sotiropoulos^1,4^

^1^ Oxford Centre for Functional Magnetic Resonance Imaging of the Brain (FMRIB), University of Oxford, UK

^2^ Department of Computer Science & Centre for Medical Image Computing, University College London, UK

^3^ Department of Neuroscience, Washington University, St. Louis, MO, US

^4^ Sir Peter Mansfield Imaging Centre, School of Medicine, University of Nottingham, UK

**Supplementary material**

*Spherical harmonics basis function*

In the present work we use the following equation to expand a spherical function using spherical harmonics:

$$f\left( \theta,\phi\right)=\sum_{l=0}^{l_{max}} \sum_{m=-l}^{l} c_{l}^{m}Y_{l}^{m}\left( \theta,\phi\right)$$

with

$$Y_{l}^{m}\left( \theta,\phi\right)=\sqrt{\frac{2l+1}{4\pi}\frac{\left( l-m \right)!}{\left( l+m \right)!}}P_{l}^{m}\left( cos\theta\right)e^{im\phi}$$

where *f* is the original function, $c_{l}^{m}$, $Y_{l}^{m}$ and $P_{l}^{m}$ are, respectively, the spherical harmonic coefficients, spherical harmonic functions and associated Legendre polynomials of order *l* and degree *m* up to the maximum order *l_max_*.

Matrix ***B*** in Eqs. 2 and 3 projects the spherical harmonics coefficients of the fod onto a set of *M* = 252 amplitudes on the sphere (obtained by a sphere geodesic tessellation). We follow the same formalism as previous works (Descoteaux et al., 2007; Tournier et al., 2007; Tournier et al., 2004) to define such matrix by computing it as follows:

$$\boldsymbol{B}_{i,j}=\left\{ \begin{matrix} Re\left( Y_{l}^{m}\left( \theta_{i},\phi_{i} \right) \right) for m>0 \\ Y_{l}^{0} for m=0 \\ Im\left( Y_{l}^{m}\left( \theta_{i},\phi_{i} \right) \right) for m<0 \end{matrix} \right.$$

with

$$l,m\mathbb{\in N;-}l\leq m\leq l$$

$$j=1+m+\sum_{t=1}^{l} 2t$$

where $Y_{l}^{m}$ are the spherical harmonic functions of order *l* and degree *m* up to the maximum order *l_max_* and *i* is the i^th^ sampled orientation on the tessellated sphere.

Matrix ***C*** in Eqs. 2 and 3 represents the spherical convolution matrix that maps the fod coefficients onto the set of sampled diffusion orientations (Tournier et al., 2007; Tournier et al., 2004). It is obtained by the product of two matrices:

$$\boldsymbol{C}=\boldsymbol{QR}$$

where ***Q*** is a matrix that maps the spherical harmonics coefficients onto the set of acquired diffusion encoding orientations and ***R*** is the matrix of rotational harmonics coefficients of the response function. Matrix ***Q*** can be obtained by using the same definition employed for matrix ***B*** and considering only even harmonic orders. Matrix ***R*** is diagonal and its elements can be computed as follows:

$$\boldsymbol{R}_{i,i}=r_{l}$$

where *r_l_* is the ratio between the spherical harmonic coefficient of order *l* and degree *m* = 0 of the response function and the corresponding coefficient of a delta function and:

$$i=0.5l\left( l+1 \right)+m$$

**Supplementary figures and legends**


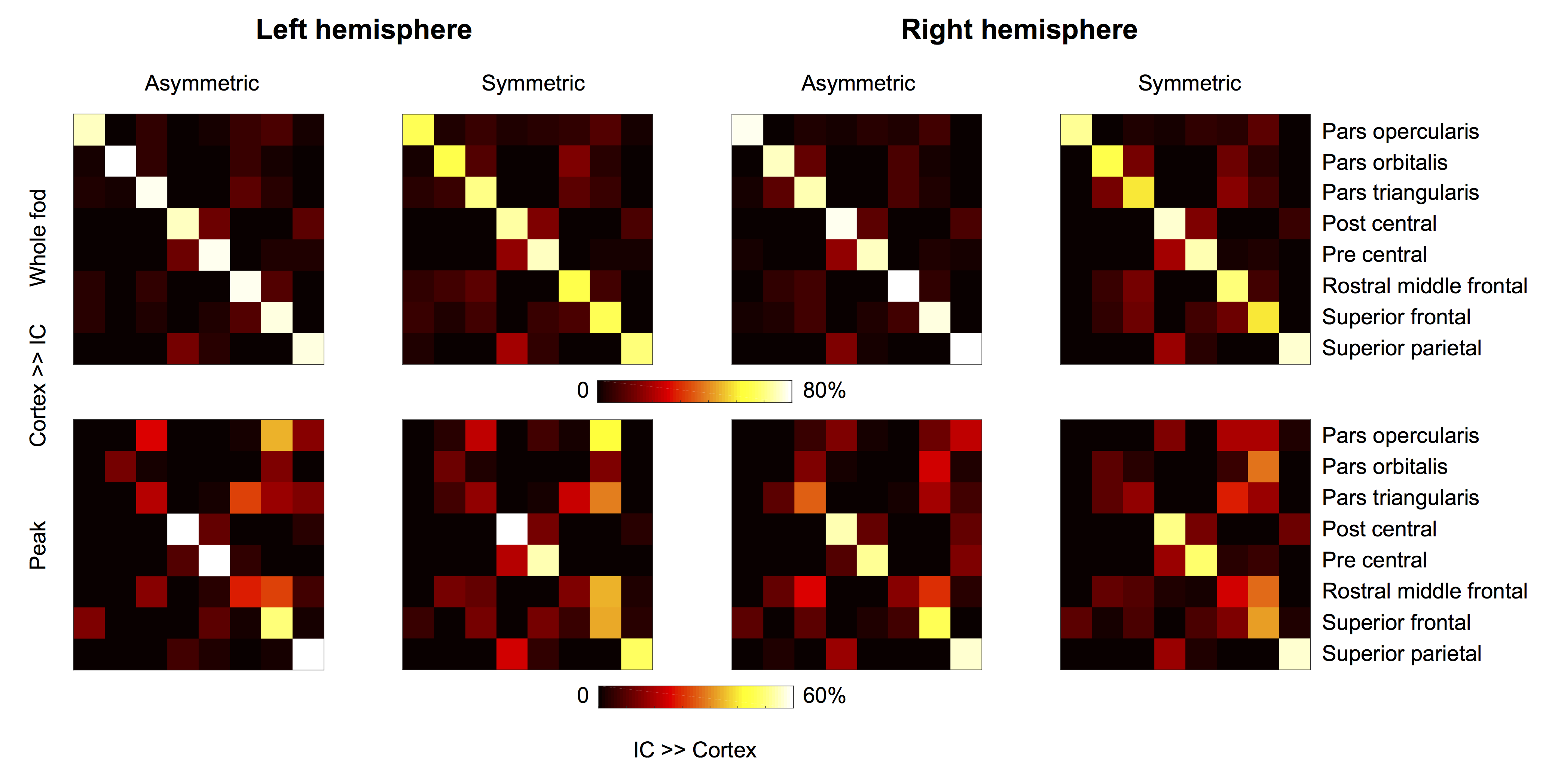


**Supplementary figure 1:** Results of the topography preservation experiment performed on in vivo data using a local whole-fod (top row) and peak-based (bottom row) tractography approach. The experiment was run in both hemispheres. Such experiment is designed to test the improvement for the whole-fod approach and we do not expect major differences for the peak-based approach. That is because peak-based approaches do not take into account fibre dispersion, i.e., peaks at the opposite sides of a fanning fod will be the same irrespective of the polarity. The matrices show the averaged (n = 10 subjects) row-normalised streamline counts that project back to a patch in the IC, which was preferentially projecting to the same cortical area. Using the proposed asymmetric fods-based tractography algorithm, more streamlines project back to their original cluster within the internal capsule. This is shown by higher values on the matrix diagonal.


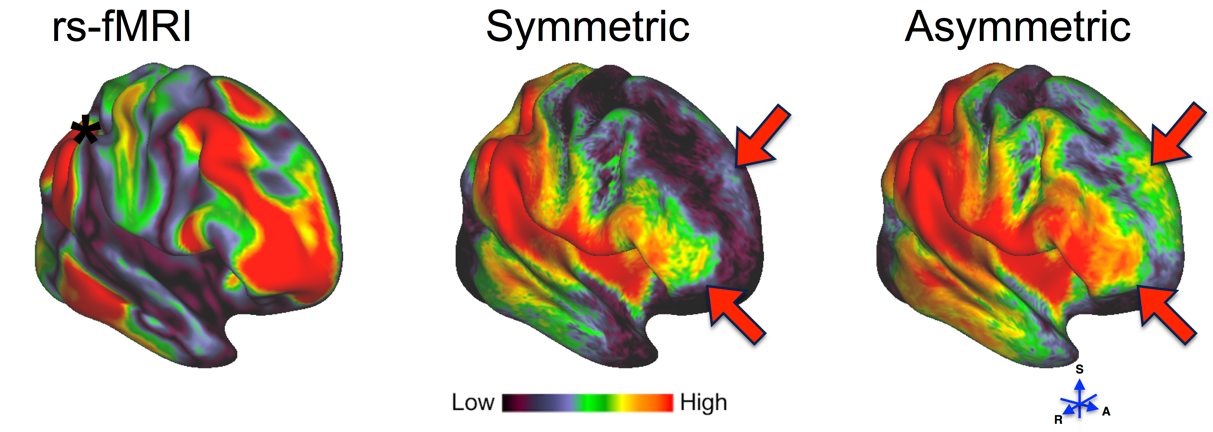


**Supplementary figure 2:** Seed-based functional (first panel) and structural (second and third panels) connectivity maps overlaid on an inflated right hemisphere surface. Black asterisk identifies the TPJ seed region. Red arrows point at cortical areas with obvious qualitative differences in connectivity strength between symmetric and asymmetric whole-fod-based tractography. To aid visualisation, the functional connectivity map shows the absolute value of the fMRI time-series correlation. The *partial* correlations between the functional connectivity and the structural connectivity profiles obtained using A-fod (S-fod) *peak*-based tractography (not shown), after regressing out the S-fod (A-fod) tractography results are r_left_ = 0.01 and r_right_ = 0.04 (r_left_ = -0.01, r_right_ = -0.02).

**References**

Descoteaux, M., Angelino, E., Fitzgibbons, S., Deriche, R., 2007. Regularized, fast, and robust analytical Q-ball imaging. Magn Reson Med 58, 497-510.

Tournier, J.D., Calamante, F., Connelly, A., 2007. Robust determination of the fibre orientation distribution in diffusion MRI: non-negativity constrained super-resolved spherical deconvolution. Neuroimage 35, 1459-1472.

Tournier, J.D., Calamante, F., Gadian, D.G., Connelly, A., 2004. Direct estimation of the fiber orientation density function from diffusion-weighted MRI data using spherical deconvolution. Neuroimage 23, 1176-1185.
